# Evaluation of the use of therapeutic peptides for cancer treatment

**DOI:** 10.1186/s12929-017-0328-x

**Published:** 2017-03-21

**Authors:** Susan Marqus, Elena Pirogova, Terrence J. Piva

**Affiliations:** 1 School of Engineering, RMIT University, Bundoora, VIC 3083 Australia; 2 School of Health and Biomedical Sciences, RMIT University, Bundoora, VIC 3083 Australia

**Keywords:** Cancer, Therapeutic peptides, Cell penetrating peptides, Apoptosis

## Abstract

Cancer along with cardiovascular disease are the main causes of death in the industrialised countries around the World. Conventional cancer treatments are losing their therapeutic uses due to drug resistance, lack of tumour selectivity and solubility and as such there is a need to develop new therapeutic agents. Therapeutic peptides are a promising and a novel approach to treat many diseases including cancer. They have several advantages over proteins or antibodies: as they are (a) easy to synthesise, (b) have a high target specificity and selectivity and (c) have low toxicity. Therapeutic peptides do have some significant drawbacks related to their stability and short half-life. In this review, strategies used to overcome peptide limitations and to enhance their therapeutic effect will be compared. The use of short cell permeable peptides that interfere and inhibit protein-protein interactions will also be evaluated.

## Background

Cancer is the second most common cause of death, being responsible for 8.2 million deaths worldwide in 2013 [1, 2]. Of particular interest is that in industrialised countries the incidence of cancer is much higher than that seen in less developed countries [2]. The incidence in these less developed countries is expected to rise due to the growth and ageing of their populations along with an increase in the prevalence of known risk factors [3]. In males, lung cancer is the leading cause of cancer worldwide deaths, while for women this is the case only in industrialised countries, while in less developed countries breast cancer is the leading cause of cancer deaths [1, 2]. Cancer has been characterised by the mutations of somatic genes that alter the function of the protein(s) they encode for [4]. Somatic alterations have been observed in most solid tumours such as those of the colon, breast, brain and pancreas. Nearly all (95%) of these altered mutations are single base substitutions, while the other 5% result from the insertion or deletion of one or a few base pairs [4]. Cancer is not a single disease with more than 100 different types known [5]. There is an extensive heterogeneity present between the same type of tumour in different individuals (intertumour heterogeneity) and among cancer cells within the same tumour (intratumour heterogeneity) [6]. Primary tumours are genetically heterogeneous and consist of multiple subpopulations of cancer cells which differ with respect to genotype(s) and phenotype(s) [7].

In the recent years, there has been a significant progress; in the diagnosis, treatment and prevention of some types of cancer [8]. Currently cancer treatments, involve surgery, chemotherapy, radiation, biological and hormonal therapy. However, the main problems with these current treatments are their high cost and adverse side effects [9]. Doxorubicin is a conventional chemotherapeutic agent that is widely used in the treatment of many tumours. However, it causes oxidative stress-mediated injury to the kidney [10], heart [11], and brain [12]. Metastatic breast cancer resistance to chemotherapeutic agents remain an obstacle for effective treatment. Chemotherapeutic agents such as taxanes and anthracyclines are not effective in controlling breast cancer as this tumour develops resistance to such drugs [13]. The overall median survival of patients with brain metastases from breast cancer was found to be 8.3 months [14]. Even if initial conventional cancer treatments are successful, the risk of tumour recurrence remains a challenge [15]. In this review, we will focus on using therapeutic peptides as anti-cancer agents.

## Therapeutic peptides

Peptides are short linear chains of amino acids (AA). They are usually <50 AA in length and are often stabilised by disulfide bonds [16]. They are designed by rational methods with high specificity to bind and modulate a protein interaction of interest. Many sequences, structures and pattern interactions of oncogenic proteins are available; and as such peptides can be designed specifically as an inhibitor of these interactions [17] – for example, if an interaction of two proteins is known, a peptide can inhibit this interaction provided if the sequence of the binding site is known [18]. If a protein-protein interaction site is unknown, a series of overlapping peptides of the desired protein are synthesised and can be tested for their capability to bind and inhibit this target interaction [19]. The peptide sequence can also be modulated easily, due to their ease of synthesis either by chemical or molecular biological techniques [17].

Therapeutic peptides have several important advantages over proteins or antibodies: they are small in size, easy to synthesise and have the ability to penetrate the cell membranes. They also have high activity, specificity and affinity; minimal drug-drug interaction; and biological and chemical diversity. An added benefit of using peptides as a treatment is that they do not accumulate in specific organs (e.g. kidney or liver), which can help to minimise their toxic side effects [20]. They can also be rapidly synthesised and easily modified [21] and are less immunogenic than recombinant antibodies or proteins [22]. Therapeutic peptides show great potential in the treatment of many diseases (for further information see [23]). In the case of cancer, these peptides can be used in a variety of ways, including carrying cytotoxic drugs, vaccines, hormones and radionuclides [24]. However, therapeutic peptides do have some significant drawbacks such as their stability in vivo. They have little or no resistance to cleavage by serum proteases in vivo [22] as well as a short half-life, low bioavailability, and production and manufacturing challenges [20]. Some of important advantages and disadvantages of using therapeutic peptides as drugs [25] are shown in Table 1.

**Table 1:** Advantages and disadvantages of therapeutic peptides (Adopted from [25])

Advantages	Disadvantages
High potency of action	Metabolic instability
High target specificity and selectivity	Poor membrane permeability
Wide range of targets	Poor oral bioavailability
Low toxicity	Poor solubility
Fewer side effects	Rapid clearance
Low accumulation in tissues	High manufacturing cost
High biological and chemical diversity	Poor activity

**Table 2:** Therapeutic peptides and their uses

Peptide name	Validation	Cell lines examined^a^	Ref.
Antimicrobial peptides			
Magainin II	in vitro	Bladder cancer cells: RT4 pathologic grade 1, 647 V grade 2, and 486P grade 4	[32]
NRC-3 and NRC-7	In vitro & in vivo	Breast cancer: MDA-MB-231, MDA-MB-468, SKBR3, MCF-7 and paclitaxel resistant MCF-7 (MCF-7-TX400) and murine mammary 4 T1 carcinoma cells	[34]
Buforin IIb	In vitro & in vivo	Cervical carcinoma (HeLa), leukaemia (Jurkat cells) and lung cancer (NCI-H460) cells	[37]
BR2	in vitro	Cervical carcinoma (HeLa), colon cancer (HCT116) and murine melanoma (B16-F10) cells	[52]
Cell penetrating peptides			
Dox-TAT	in vitro	Breast cancer (MCF-7 and MCF-7/ADR) and rat prostate carcinoma (AT3B1) cells	[53]
Tumour targeting peptides			
RGD-SSL-Dox	In vitro & in vivo	Melanoma (A375) and murine (B16-F10) melanoma cells	[61]
LPD-PEG-NGR	In vitro & in vivo	Fibrosarcoma (HT-1080) cells	[66]
Therapeutic peptides target transduction pathway			
PNC-2 and PNC-7	in vitro	Pancreatic cancer (MIA-PaCa) cells	[109]
Cardiac natriuretic peptides	In vitro & in vivo	Pancreatic cancer (HPAC), renal carcinoma (SW156), breast adenocarcinoma (HCCI428), ovarian adenocarcinoma (NIHOVCAR-3), modularly thyroid carcinoma (TT), glioblastoma (LNZTA3WT4) and lung carcinoma (NCI-H1963) cells	[111–118]
RGD-PEG-Suc-PD0325901	In vitro & in vivo	Glioblastoma (U87MG) cells	[126]
VWCS	In vitro	Head and neck squamous cell carcinoma (HNSCC) and oral epidermoid carcinoma (KB) cells	[140]
FWCS	In vitro	Head and neck squamous cell carcinoma (HNSCC) and oral epidermoid carcinoma (KB) cells	[141]
Therapeutic peptides target cell cycle			
p16	In vitro	Pancreatic cancer (AsPC-1 and BxPC-3) cells	[166]
Bac-7-ELP-p21	In vitro	Ovarian carcinoma (SKOV-3) cells	[75]
Pen-ELP-p21	In vitro	Cervical carcinoma (HeLa) and ovarian carcinoma (SKOV-3) cells	
Therapeutic peptides induce cell death			
TAT-Bim	In vitro & in vivo	Murine T-cell lymphoma (EL4), pancreatic cancer (Panc-02) and melanoma (B16-F10) cells	[193]
Poropeptide-Bax	In vitro	Melanoma (SK-MEL-28) cells	[194]
R8-Bax	In vitro & in vivo	Cervical carcinoma (HeLa) and murine mammary carcinoma (TS/A) cells	[194]
CT20p-NP	In vitro & in vivo	Breast cancer (MCF-7 or MDA-MB-231) and colon cancer (HCT-116) cells	[195]
RRM-MV	In vitro	Squamous cell carcinoma (COLO16) and malignant melanoma (MM96L), and murine melanoma (B16-F10) cells	[197, 199]
RRM-IL12	In vitro	Mouse melanoma (B16-F10) cells	[197]
Therapeutic peptides target tumour suppressor protein			
PNC-27	In vitro	Cervical carcinoma (HeLa), colon cancer (SW1417 and H11299), breast cancer (MDA-MB-453 and MCF-7), osteosarcoma (SAOS2), leukaemia (K562), pancreatic cancer (MIA-PaCa-2) and melanoma (A-2058) cells. Rat k-ras-transformed pancreatic cancer (TUC-3) and transformed endothelial (E49) cells	[214, 216, 217]
PNC-21	In vitro	Cervical carcinoma (HeLa), colon cancer (SW1417 and H1299), breast cancer (MDA-MB-453), and osteosarcoma (SAOS2) cells. Rat k-ras-transformed pancreatic cancer (TUC-3) and transformed endothelial (E49) cells	[214]
PNC-28	In vitro & in vivo	Breast cancer (MDA-MB-453), colon cancer (H1299 and SW1417), osteosarcoma (SAOS2), cervical carcinoma (HeLa) and pancreatic cancer (MiaPaCa-2) cells. Rat k-ras-transformed pancreatic cancer (TUC-3) and transformed endothelial (E49) cells	[214, 219, 232]
Tat-αHDM2	In vitro & In vivo	Melanoma (MM-23, MM-24 and MM-26), retinoblastoma (Y79 and WERI), osteosarcoma (U2OS), and cervical carcinoma (C33A) cells	[220]
Therapeutic peptides target transcription factors			
Int-H1-S6A, F8A	In vitro	Breast cancer (MCF-7) cells	[230]
Pen-ELP-H1	In vitro	Breast cancer (MCF-7) cells	[76]
BACl-ELP-H1	In vivo	Glioma (U-87 MG and D54) and murine glioma (C6) cells	[231]

^a^Unless specified otherwise, all cell lines are human in origin

## Peptides for cancer therapy

Therapeutic peptides are a novel and promising approach for the development of anti-cancer agents [23, 26]. Boohaker et al. [21] has classified existing therapeutic peptides for the treatment of cancer into three main groups: (a) antimicrobial/pore forming peptides, (b) cell-permeable peptides and (c) tumour targeting peptides. The cellular targets of some of these peptides are seen in Table 2.

AMP/pore-forming peptides are peptides that occur naturally in all living organisms and have specific biological activities [16]. They are part of the innate immune defence mechanism [27] and show potential as antimicrobial therapeutic agents (e.g., defensins and cathelicidins) [28]. Many of these antimicrobial peptides (AMPs) are short, possess cationic charges, and form amphipathic structures in non-polar solvents [29]. They bind to negatively charged bacterial cell membranes via electrostatic interactions, disrupting their function [29], resulting in the death of these prokaryotes [30].

These pore-forming peptides target cancer cell membranes, and can induce cell death either by necrosis or apoptosis. In necrosis, the AMPs target the negatively-charged molecules on the cancer cell membrane and cause cell lysis; while in apoptosis, they cause disruption of the mitochondrial membrane [21]. Another AMP is magainin, which is derived from the skin of the African clawed frog *Xenopus laevis* [31]. Lehmann et al. [32] observed that margainin II was cytotoxic to human bladder cancer cells but not human or murine fibroblasts. Magainin killed the bladder cancer cells by inducing pores in the plasma membrane [32]. Pleurocidin is isolated from the winter flounder, *Pleuronectes americanus* [33] and members of this family of cationic peptides (such as NRC-3 and NRC-7) were cytotoxic against human breast cancer cells and mouse mammary carcinoma cells but not human dermal fibroblasts [34]. These two peptides were shown to disrupt the integrity of the cell membrane [34]. The pre-treatment of human breast cancer cells (MDA-MB-231) with NRC-3 or NRC-7 and cisplatin enhanced the latter’s cytotoxic effect (EC_50_) by 5.5- and 1.7-fold, respectively [34].

Buforins are peptides derived from the stomach of *Bufo bufo gargarizans* [35]. Buforin I is a 39 AA peptide, from which the 21 AA buforin II is derived. Both peptides exhibit antimicrobial properties; with buforin II possessing higher activity than buforin I [36]. Buforin IIb was shown to be cytotoxic against human cervical carcinoma (HeLa) and leukaemia (Jurkat cells) cells in vitro, and suppressed the growth of human lung cancer xenografts in mice [37]. This peptide interacts with the gangliosides on the plasma membrane and induced the apoptotic extrinsic pathway in these cells [37].

The second group of therapeutic peptides are cell penetration peptides (CPPs). These peptides are 5–30 AA in length and can translocate through the plasma membrane and transport cargos ranging from small molecules (e.g., DNA, siRNA and plasmid) to oligonucleotides and proteins and as such provide a promising mechanism for drug delivery [38]. These CPPs are hydrophobic in nature and are mainly composed of basic residues, and play an important role in the interaction and insertion of peptides into the cell membrane [17]. They are taken up by the cell either by an energy-independent (direct translocation) [39] or energy-dependent (endocytosis and pinocytosis) process [40, 41]. The internalisation of these peptides depend on several factors including the size of the transported cargo [39], temperature [42], peptide concentration [42, 43] and cell type [44]. An example of a CPP is the trans-activator of transcription (Tat). The Tat peptide is derived from the human immunodeficiency virus (HIV) and is easily able to cross the cell membrane [45]. Intracellular cargos carried by this peptide across the plasma membrane include antisense oligonucleotides [46], liposomes [47], therapeutic agents [48], small interfering RNA (siRNA) [49, 50] and nucleic acids [51]. Recently Lim et al. [52] designed a novel CPP called BR2 which is 17 AA peptide based on the CPP motif of buforin IIb. This peptide was cytotoxic against HeLa cells, HCT116 human colon cancer cells and B16-F10 mouse melanoma cells but not NIH 3 T3 mouse fibroblasts, HaCat human keratinocytes and BJ human fibroblasts [52]. BR2 was shown to interact with gangliosides on the cell membrane of thee tumour cells [52]. Doxorubicin conjugated to the Tat peptide was taken up by drug resistant tumour cells such as human breast cancer (MCF-7 and MCF-7/ADR) and AT3B1 rat malignant prostate cells resulting in their death [53].

The third group of peptides are the tumour-targeting peptides (TTPs). These peptides target markers such as receptors expressed on the tumour cell membrane [21]. RGD contains the sequence Arg-Gly-Asp which recognises and binds to integrin ανβ3 and ανβ5 [54] expressed on the membrane of lung cancer [55], melanoma [56], brain tumours [57], ovarian carcinoma [58] and breast cancer cells [59]. This peptide (RGD) could be used as a drug delivery system due to its ability to be internalised into the cell [60]. Xiong et al. [61] fused the RGD peptide onto the surface of sterically stabilised liposomes (SSL) (a modified liposome with hydrophobic polymer (PEG) on its membrane [62]) loaded with doxorubicin. As a result, RGD-SSL-Dox enhanced doxorubicin’s efficacy against murine B16-F10 and human A375 melanoma cells grown in vitro as well as B16-F10 tumours grown in vivo in mice [61].

Another TTP peptide is NGR, which contains a Asn-Gly-Arg sequence. NGR binds to aminopeptidase N (APN) (also known as CD13) which is highly expressed by endothelial tumour cells such as scirrhous gastric cancer [63], pancreatic cancer [64] and non-small cell lung carcinoma [65]. Chen et al. [66] fused NGR peptides to PEGylated LPD (liposome-polycation-DNA) nanoparticles for the intracellular delivery of either c-myc siRNA and/or doxorubicin in HT-1080 human fibrosarcoma cell xenografts. The c-myc siRNA inhibited the expression of the c-myc protein and induced apoptosis in HT-1080 cells grown both in vitro and in vivo [66]. The co-delivery of siRNA and doxorubicin significantly inhibited the growth of HT-1080 xenografts in mice [66].

## Strategies to overcome peptide limitations

Recently, many mechanisms have been developed to overcome the limitations of these therapeutic peptides. In order to overcome their poor cell permeability; various CPPs have been used to enhance the intracellular delivery of peptides [67]. The Tat peptide was conjugated with multiple pro-apoptotic peptides (KLAKLAK)_2_ separated by a caspase-3 cleavage site [68]. When this peptide was taken by mouse melanoma and human breast cancer cells, it activated endogenous caspase-3 which then cleaved the Tat-KLA peptide resulting in release of the pro-apoptotic peptide (KLAKLAK)_2_ [68]. This peptide induced apoptosis in these cells in vitro as well as inhibiting the growth of mouse melanoma xenografts in mice [68]. This peptide induced apoptosis in the numerous cancer cell lines such as non-small cell lung carcinoma (A549), cervical carcinoma cells (HeLa), breast cancer cells (MCF-7), and malignant melanoma cells (A375) [69]. To enhance the selectivity and specificity of Tat-KLA peptide for tumour cells, it was conjugated to the BRBP1 peptide (12 AA) which was previously identified to possess affinity for human breast cancer cells MDA-MB-231 that metastasize to the brain and where they were renamed 231-BR cells [70]. The BRBP1-Tat-KLA peptide significantly reduced the viability and migratory capacity of 231-BR cells grown in mice and was cytotoxic to human breast cancer subtypes (BT-474 and MDA-MB-231) [71].

Elastin-like polypeptides (ELPs) are biopolymers that contain a pentapeptide (Val-Pro-Gly-Xaa-Gly) repeat sequence. These biopolymers are sensitive to temperature and undergo a phase transition from soluble to insoluble and form aggregates at 40-42 °C [72]. ELP has been used for various delivery systems such as small drug molecules [73], peptides [74–76], antibodies [77], proteins [78] and plasmid DNA [79]. It has the potential to target the delivery of peptides or drugs to solid tumours [80]. Meyer et al. [81] have shown increased ELP accumulation (~two-fold) in tumours that were locally treated with hyperthermia compared to those that were not. ELP also enhanced the delivery of ELP-conjugated therapeutic peptides, which inhibited the attachment, spreading, migration and invasion of human ovarian cancer cells in cell culture and also inhibited the metastasis of ovarian cancer cells in vivo [82].

Multidrug resistance remains one of the major obstacles of conventional cancer treatments [83]. Some anti-cancer treatments are losing their effectiveness against tumours which possess a resistant phenotype [84]. Some tumour cells develop an intrinsic resistance to chemotherapeutic drugs, whereas others only develop resistance after exposure [84]. Recently, ELPs have been used to overcome drug resistance in some tumour cells. In one study, a Tat-ELP-GFLG-Dox polypeptide consisting of a Tat peptide fused at the N-terminal, ELP, a tetrapeptide linker (GFLG) which was used to release its drug following cell entry and at the C-terminal a thiol reactive derivative of doxorubicin WP936, was able to overcome the efflux pumps in MES-SA/Dx5 and NCI/ADR-RES human uterine sarcoma cells [85]. Moreover, this polypeptide accumulated in these uterine sarcoma cells while that of free doxorubicin was pumped out of the cell. This polypeptide formed aggregates when these cells were treated for 1 h at 42 °C [85] where its cytotoxicity was enhanced ~20-fold as a result [86].

Penchala et al. [87] recently developed a new strategy for enhancing the in vivo half-life of peptides without compromising their potency. They conjugated a number of peptides to a small molecule called AG10, which binds with high affinity and selectivity to the plasma protein Transthyretin (TTR) [87]. Conjugating peptides to AG10, through short linkers, allowed these peptides to bind reversibly to TTR in human plasma. By recruiting the bulk of TTR (56 kDa protein), they were able to protect the peptides against proteases in serum and also decrease the filtration of the peptides though the kidneys [87]. A number of peptides were used in the study including tripeptide (Arg-Gly-Lys-MCA), neurotensin (13 AA neuropeptide), native gonadotropin-releasing hormone (GnRH: 10 AA peptide hormone) and D-6-Lys-GnRH (GnRH agonist: 10 AA). The half-life of the native GnRH was at least 13-fold longer than its non-native agonist [87]. Evaluation of the D-6-Lys-GnRH conjugate in male rats showed that binding to TTR extended its circulatory half-life which resulted in enhanced efficacy as measured by elevated levels of circulating testosterone [87].

PEGylation is another approach to increase the half-life of peptides. It involves conjugating polyethylene glycol (PEG) to macromolecules such as proteins or peptides; increasing the size of a polypeptide and reducing its renal filtration and clearance [88]. However, the problem with PEG or PEG-conjugated macromolecules is that it induces the production of antibodies against PEG in healthy individuals exposed to PEG-containing compounds (e.g., cosmetics, pharmaceuticals and processed food) or in patients treated with PEG-conjugated agents [89]. Therefore, a new recombinant polypeptide has been developed termed XTEN. It is 864 AA in length and mimics PEG, and is a stable, soluble protein and has reduced immunogenicity [90]. The conjugation of XTEN into a protein or peptide resulted in an increase in their half-life [90, 91]. Teduglutide is a glucagon-like peptide-2 (GLP2) analogue that has a half-life of ~ 3–5 h in human serum [92] when conjugated with XTEN, its half-life increased in mice, rat and monkey serum by 34, 38 and 120 h, respectively [93].

Xiao et al. [94] conjugated site-specific modified betaine onto the amino-terminal end of bacterial xanthine guanine phosphoribosyltransferase (CG-GPRT) and the HIV inhibitory peptide CG-T20. Betaine reduced the aggregation and increased the solubility of both the protein CG-GPRT and the peptide conjugated CG-T20 [94]. Hao et al. [95] fused the Tat peptide into the C-terminal of HPRP-A1 α-helical (anti-cancer peptide) which is derived from *Helicobacter pylori*. The fusion of Tat peptide into HPRP-A1 peptide significantly increased its uptake compared to that of the unfused peptide. The Tat peptide was shown to protect HPRP-A1 peptide from degradation as well as exerting specific anticancer activity against human cervical carcinoma cells [95].

## Therapeutic peptides target signal transduction pathways

**Fig. 1:**
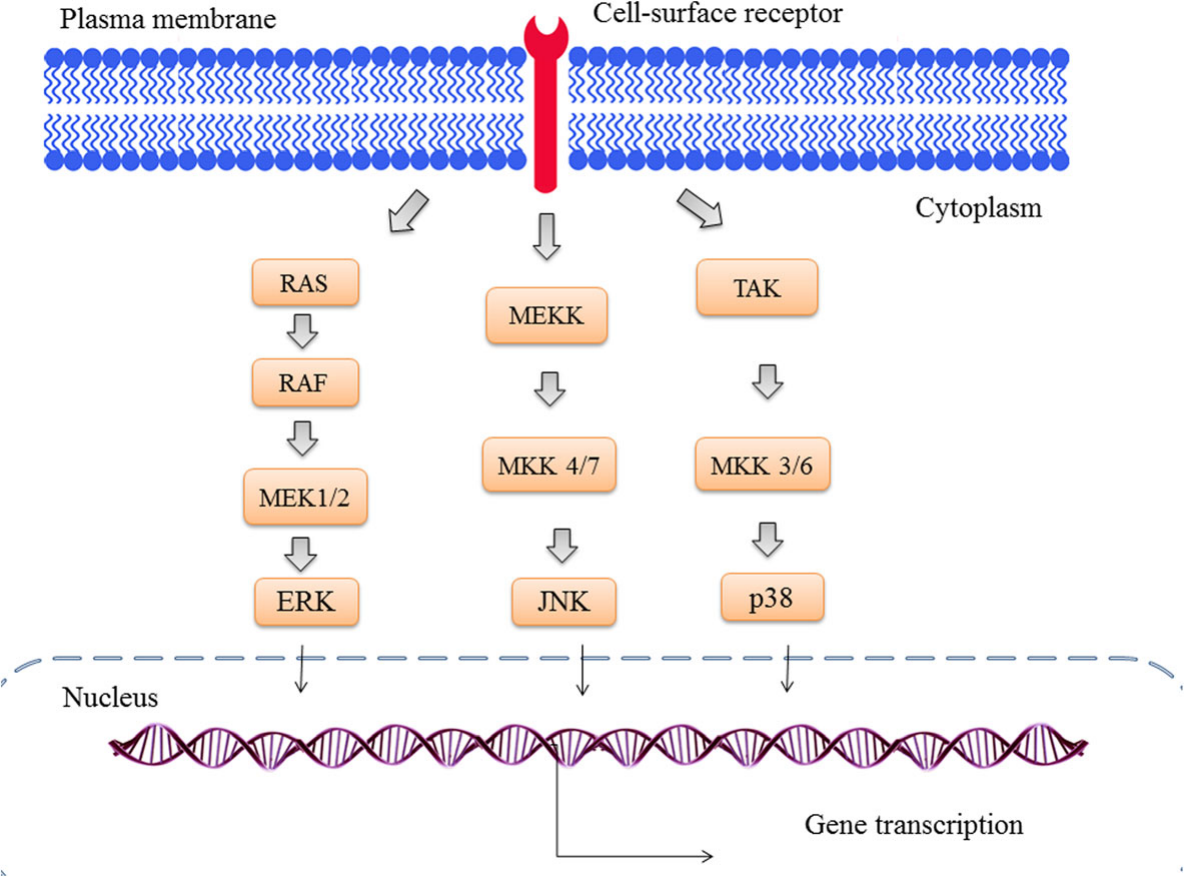
MAPK signalling pathways (Adopted from [100])

Bidwell et al. [17] have classified therapeutic peptides into three main groups, based on their biological targets: (a) signal transduction pathways, (b) cell cycle regulation and (c) cell death pathways [96]. Many therapeutic peptides have been designed to inhibit mitogen-activated protein kinases (MAPKs). MAPKs are serine/threonine kinases and have an important role in cellular signal transduction cascades and trigger intracellular events in response to external growth factors, hormones, nutrient status or stress [97]. These signals are transmitted into the nucleus resulting in changes in gene expression [98]. Constitutive activation of the MAPK pathway has been observed in pancreatic, colon, lung, ovarian and kidney tumours [99]. The three main characterised subfamilies of MAPKs found in mammalian cells include the extracellular signal-regulated kinases (ERKs), c-Jun amino terminal kinases (JNKs) and p38 MAPKs (Fig. 1) [100]. ERK plays an important role in cellular proliferation and differentiation [101] and is deregulated in one-third of all human cancers such as breast, pancreatic, lung adenocarcinoma, thyroid, bladder, liver, kidney and melanoma [102].

Developing small molecule inhibitors that target the Ras/Raf/MEK/ERK pathway has great potential for cancer therapy [103]. There are three Ras isoforms expressed in mammalian cells: H-Ras, K-Ras and N-Ras [104] and they control signalling pathways that are responsible for cell growth and malignant transformation [105]. Oncogenic mutations of Ras are found in many human cancers derived from the skin, cervix, ovary, urinary tract, stomach, lung, breast, prostate, thyroid, and large intestine [104]. The Ras gene encodes for a p21 protein which becomes oncogenic and causes malignant transformation of cells when a single substitution base mutation occurs in glycine at position 12 and glutamine at position 61 [106]. The ras-oncogene p21 peptide, called PNC-7 is 35–47 residues long and is derived from the GAP-binding region of p21 found to inhibit cell transformation [107]. Kanovsky et al. [108] found that two ras-p21 peptides PNC-7 and PNC-2 (96–110 residues) induced phenotypic reversion of both ras-transformed rat pancreatic cancer cells (TUC-3) to their untransformed phenotypes. These peptides were linked to penetratin a CPP; which induced the phenotypic reversion of ras-transformed human fibrosarcoma cells (HT-1080) to its untransformed phenotype [109]. Both peptides induced the death of MIA-PaCa-2 human pancreatic cancer cells by inhibiting ras-p21 phosphorylation [109], however it did not cause these cells to undergo phenotypic reversion.

In animals, peptides that possess anti-cancer activities are mainly found in the immune system, central nervous system, digestive system, heart, bone, muscle and skin [110]. Vesely et al. [111–118] investigated four types of cardiac natriuretic peptides, including atrial natriuretic peptide (ANP), vessel dilator peptide, long-acting natriuretic peptide (LANP) and kaliuretic peptide. These four peptides normally circulate in the human body and have shown anti-cancer activity against human cancers, including: pancreatic, breast, prostate, renal, colon, ovarian, melanoma, brain, thyroid and lung. Sun et al. [119] observed that these four peptide hormones inhibited the activation of ERK1/2 by epidermal growth factor (EGF) and insulin in human prostate adenocarcinoma and pancreatic cancer cells. Moreover, the vessel dilator and kaliuretic peptide exhibited anti-cancer properties against human prostate carcinoma by inhibiting Ras activity [120]. Sun et al. found that LANP and ANP significantly inhibited (80-90%) MEK1/2 and ERK1/2 activity in human prostate adenocarcinoma cells [121–123].

MEK plays an important role in phosphorylating ERK that amongst its roles induces the transcription of proteins that control apoptosis [124]. Li et al. [125] developed a series of conjugates consisting of the RGD peptide fused to a MEK inhibitor derived from a small molecule of the MEK1/2 inhibitor PD0325901. The RGD-MEKI conjugates inhibited ERK1/2 signalling and halted melanoma A375 cells at the G1 cell cycle checkpoint in an analogous manner to that of PD0325901 [125]. Hou et al. [126] fused the RGD peptide with PD0325901 and a PEG-linker and this RGD-PEG-Suc-PD0325901 peptide was very efficient in targeting human glioblastoma U87MG αvβ3 receptor positive cells by blocking ERK1/2 signalling and inhibiting metastasis of these xenografts grown in mice [126].

The second subfamily of MAPKs signalling molecules is JNK1/2. JNK1/2 activity is elevated in many cancers including those from the colon [127], pancreas [128], breast [129], blood [130], head and neck [131], stomach [132], and brain [133]. JNK peptide inhibitors, derived from the JNK interacting protein (JIP), were conjugated into the N-terminal region of the inverted Tat peptide (JIP^10^-Δ-TAT^i^) or conjugated into a peptide consisting of nine arginines (JIP^10^-Δ-R_9_) [134]. These JIP peptides inhibited JNK2 activity and were 10-fold more selective in binding to his isoform rather than to JNK1 or JNK3 and were shown to inhibit the metastasis of murine mammary cancer cells in vivo [134].

The third MAPK subfamily member is p38. Levels of p38α are elevated in many cancers such as head and neck squamous cell carcinoma (HNSCC) [135], breast [136], gastric [137] and non-small cell lung cancer [138]. It is composed of two binding sites; an ATP-binding site and a Asp-Phe-Gly (DFG) motif [139]. Recent studies have focused on design peptides that are inhibitory to p38α. The synthetic tetrapeptide VWCS, based on the ATP-binding site, inhibited p38α activity in HNSCC [140]. This peptide bound to the ATP binding site of p38α and inhibited the proliferation of HNSCC and KB human oral cancer cells in a time- and dose- dependent manner [140]. The FWCS tetrapeptide is based on the DFG site and inhibited the growth of HNSCC and KB cells in a dose- and time-dependent manner [141].

## Therapeutic peptides that target the cell cycle

**Fig. 2:**
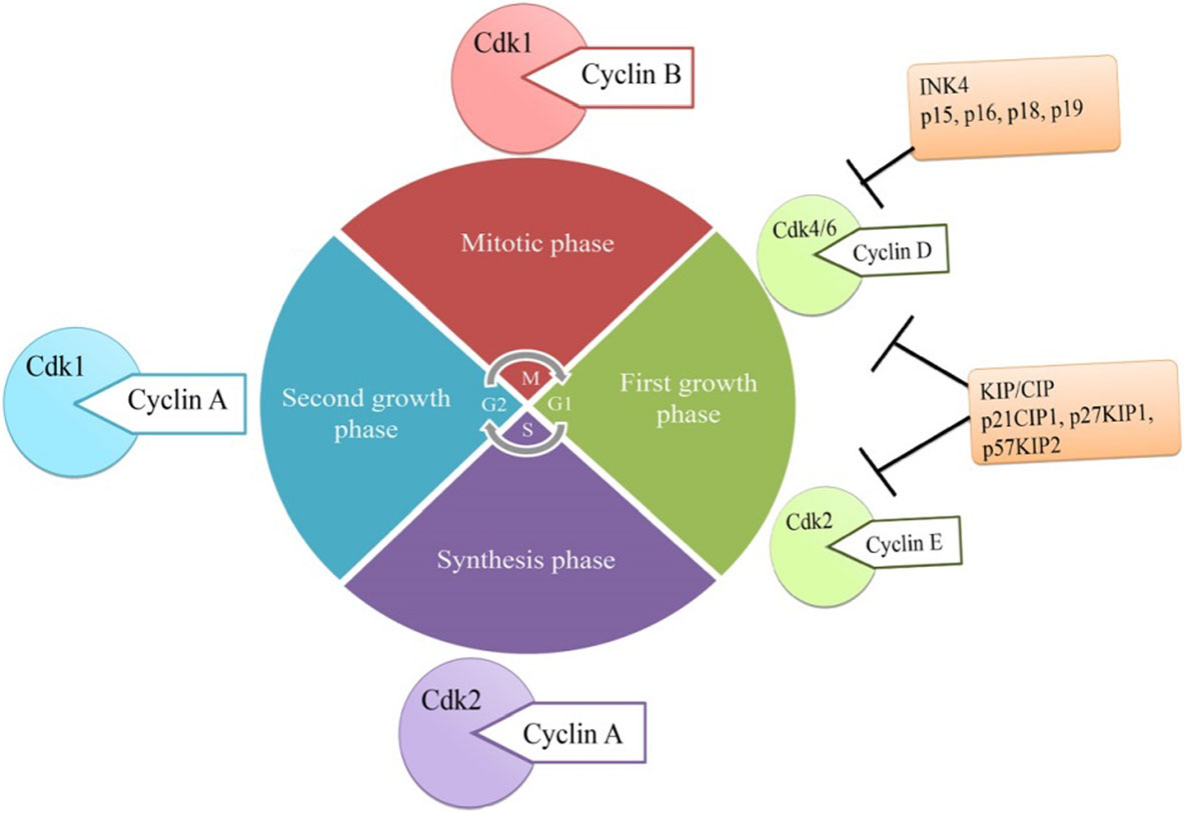
Cell cycle in eukaryotic (adopted from [147])

The cell cycle is composed of four distinct phases, including two gap phases G1 and G2 as well as S-phase and M-phase [142]. The progression of a cell through the different phases of the cell cycle is under the control of several classes of cyclin-dependent kinases (Cdks). Cdks belong to a large family of catalytic subunits of heterodimeric serine/threonine protein kinases [143]. In mammalian cells, as the cells progresses from G1 to mitosis a number of kinase subunits are expressed. In G1 phase, Cdk4 and Cdk6 in association with cyclin-D play a vital role in the cell progressing during this phase [144]. The cyclin E-Cdk2 complex regulates the movement of the cell from G1 into S-phase [145]. In S-phase, cyclin A-Cdk2 complexes control the progression of cells through this phase [146] while cyclin B-Cdk1 promotes the progress of the cell in mitosis (Fig. 2) [147].

The activation of Cdks in the cell is regulated by Cdk inhibitors (CKIs) [148]. There are two families of CKIs: (1) INK4 proteins which include: p16^INK4a^, p15^INK4b^, p18^INK4c^ and p19^INK4d^ and (2) CIP/KIP proteins which include; p21^cip1/waf1^, p27^kip1^ and p57 ^kip2^ (Fig. 2) [149]. The tumour suppressor protein p16 inhibits the progression of cells from G1 to S phase by binding to Cdk4/6 which prevents cyclin D from binding to this kinase [150]. The cyclin D-Cdk4/6 complex phosphorylates the retinoblastoma (pRb) protein that results in the release of the transcription factor E2F which then promotes the progression of the cell from G1 to S phase [151]. Mutations of p16 have been observed in tumours of the breast [152], prostate [153], skin [154], oropharynx [155], cervix [156], colon [157] and brain [158]. The expression of exogenous p16 in transfected cancer cells restored the activity of wild type p16 and induced apoptosis in human brain, prostate, lung and bladder cancers [159–163]. Fahraeus et al. [164] identified a 20 AA synthetic peptide of the p16 protein (84–103 AA) that bound to Cdk4/6 which inhibited the formation of the cyclin D-Cdk4 complex, as well as pRb phosphorylation. This prevented the entry of HaCat, MCF-7 breast cancer cells, MRC-5 fibroblasts, HT-29 colon carcinoma cells and 3 T3 mouse fibroblasts from entering the S-phase of the cell cycle [165]. When p16 was fused with the CPP, penetratin, it inhibited the growth of both p16 negative and pRb positive human pancreatic cancer cell lines (AsPC-1 and BxPC-3) by arresting cells in G1 phase [166]. This peptide significantly suppressed the growth of human pancreatic cancer and prolonged the survival of mice without showing severe systemic toxicity [167].

The cyclin-dependent kinase inhibitor p21 induces cell cycle arrest, inhibits DNA replication and regulates apoptosis [168]. It also inhibits cell cycle progression by two mechanisms: (a) inhibiting the activity of cyclin-Cdk complex and (b) proliferating cell nuclear antigen (PCNA) function which results in arrest at the G1 and G2 cell cycle checkpoints as well as inhibiting DNA replication [169]. Peptides derived from the p21 C-terminal region contain an inhibitory domain that inhibits the activity of PCNA [170]. Warbrick et al. [171] showed that the C-terminal region of p21 (144–151 AA) interacted with PCNA and inhibited DNA replication. Chen et al. [19] observed that a p21 peptide fragment (139–164 AA) also bound to PCNA. Pan et al. [172] showed that both p21 and a p21 peptide fragment (139–160 AA) derived from the C-terminal region inhibited DNA repair in HeLa cells induced by alkylating agents or UV radiation. Ball et al. [173] found that a p21 peptide (141–160 AA) from the C-terminal domain region inhibited cyclin-Cdk4 activity and arrested cell cycle at the G1 cell cycle checkpoint. This p21 peptide was fused with penetratin inhibited both cell proliferation and cell cycle progression in human colon cancer cells by two independent mechanisms involving cyclin-Cdk and PCNA [174]. The p21 peptide (139–164 AA) fragment that was fused into penetratin inhibited cyclin-Cdk activity and induced necrosis in human lymphoma cells [175].

Recently, the p21 peptide was conjugated into ELP and Bac-7, a CPP. The Bac-7-ELP-p21 polypeptide inhibited proliferation of human ovarian cancer cells by arresting cells at the S and G2/M phases of the cell cycle [75]. This was most likely caused by the inhibition of pRb activation. The inhibitory effects of this polypeptide were enhanced when the cells were treated with hyperthermia (42 °C). Hyperthermia induced the ELP to from aggregates that bound to the cell membrane which were internalised by endocytosis [75]. Mikecin et al. [176] used a combinational treatment of bortezomib (a proteasome inhibitor) and the carboxyl-terminal of p21 peptide fused into ELP. The synergistic treatments of bortezomib and p21-ELP-Bac polypeptide at 42 °C prevented human androgen-independent prostate cancer cells to pass through the cell cycle or proliferate resulting in higher apoptotic death than compared to cells exposed to a single treatment [176]. Massodi et al. [74] conjugated the p21 peptide into ELP and a penetratin peptide. This Pen-ELP-p21 peptide displayed anti-proliferative effect against both human cervical and ovarian carcinoma cells.

## Therapeutic peptides induce cell death

**Fig. 3:**
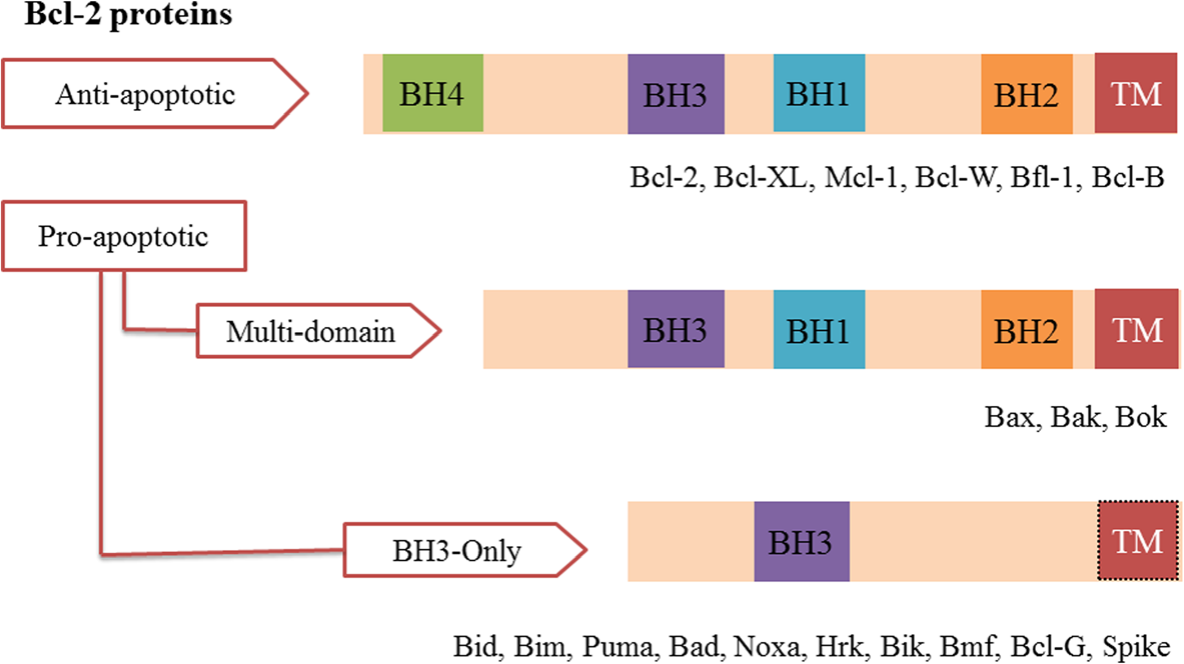
Structure represents features of Bcl-2 family proteins including anti-apoptotic and pro-apoptotic (adopted from [181])

There are two main types of cell death: apoptosis and necrosis. Apoptosis, or programmed cell death, is a normal process that plays an important role during development and ageing; it maintains stable cell populations within tissues [177]. It can also be a defence mechanism as it initiates an immune response to cell damage by diseases or noxious agents [177] and is a complex process involving different pathways [178]. When a defect occurs at any stage along this pathway, it can result in malignant transformation, tumour metastasis and resistance to cancer therapeutics [178]. The two main pathways of apoptosis are the intrinsic and extrinsic pathways. The intrinsic pathway is initiated by the release of apoptotic factors such as cytochrome c from the mitochondrial membrane and it is highly regulated by proteins belonging to the Bcl-2 family [179]. The Bcl-2 family proteins contain up to four conserved Bcl-2 homology (BH) domains (BH1, BH2, BH3 and BH4) as well as a transmembrane domain (TM). There are two main groups of Bcl-2 proteins: (a) anti- and (b) pro- apoptotic Bcl-2 proteins. The anti-apoptotic Bcl-2 proteins contain all four BH domains (BH1-BH4) and these proteins are: Bcl-2, Bcl-X_L_, Mcl-1, Bcl-W, Bfl-1, and Bcl-B (Fig. 3) [180]. The pro-apoptotic Bcl-2 proteins can be divided into multi-domain and BH3-only proteins. The multi-domain proteins contain only three BH domains (BH1, BH2 and BH3 but not BH4) such as Bax, Bak and Bok (Fig. 3). The BH3-only proteins including Bid, Bim, Puma, Bad, Noxa, Hrk, Bik, Bmf, Bcl-G and spike. They contain a limited number of BH domains but not all contain a TM domain (Fig. 3) [181].

Apoptosis is highly regulated by Bcl-2 family protein members; pro-apoptotic proteins (e.g., Bax and Bak) promote cell death whereas anti-apoptotic proteins (e.g., Bcl-2 and Bcl-X_L_) promote cell survival [182]. When a cell receives apoptotic signals initiated from stress, DNA damaging agents or infection, the BH3-only proteins activate Bax and Bak (a) either directly by binding to them or by (b) binding to anti-apoptotic proteins and indirectly activating these proteins. Once activated, the pro-apoptotic proteins Bax and Bak oligomerise and form pores in the mitochondrial outer membrane which triggers the release of cytochrome c thereby inducing the apoptotic intrinsic pathway [183]. When cytochrome c is released into the cytosol, it binds and activates apoptotic protease-activating factor-1 (APAF-1) and procaspase-9 as well, resulting in the formation of the apoptosome [184]. The apoptosome activates the initiator caspase, caspase-9 which in turn activates pro-caspase-3 and −7 and promotes apoptosis [185]. When the balance of anti-apoptotic and pro-apoptotic proteins are disrupted, it results in deregulation of apoptosis in the affected cells [178]. In cancer, the overexpression of anti-apoptotic proteins protect tumour cells from apoptosis [178] and has been observed in many human cancers including prostate [186], neuroblastoma [187], kidney [188], breast cancer [189], acute lymphoblastic leukaemia [190], chronic lymphoblastic leukaemia [191] and non-Hodgkin’s lymphomas [192].

Kashiwagi et al. [193] fused the CPP Tat peptide onto the BH3 domain derived from Bim. This Tat-Bim peptide induced apoptosis in mouse T-cell lymphoma, melanoma and pancreatic cancer cells which was enhanced when these cells were exposed to radiation [193]. Poropeptide-Bax is a peptide (106–134 AA) derived from the pore-forming domain of Bax, which induced cytochrome c release from SK-MEL-28 human melanoma cells resulting in these cells undergoing apoptosis [194]. The amino-terminal region of poropeptide-Bax peptide was fused with a poly-Arginine sequence, termed R8-Bax peptide, which induced cell death in HeLa cells in a time- and dose- dependent manner as well as the regression of TS/A-pc mice mammary carcinoma cells in vivo [194].

The CT20 peptide (CT20p) (173–192 AA) is derived from the C-terminal α-9 helix of the pro-apoptotic protein, Bax [195]. This peptide shares some similarities with AMP’s structure including: hydrophobic, cationic amino acids and two lysines [195]. CT20p encapsulated into hyperbranched polymeric nanoparticles (HBPE-NPs) disrupted the membrane integrity of human breast and colon cancer cells in vitro as well as causing the regression of human mammary adenocarcinoma xenografts grown in mice [195]. CT20p-NP was cytotoxic toward human metastatic breast cancer cells (MDA-MB-231), but not to human normal breast epithelial cells (MCF-10A) [196].

Another cell death-inducing peptide is RRM-MV [197]. RRM-MV is a synthetic peptide that has been computationally designed using the resonant recognition model (RRM). It is a short linear peptide (18 AA) that mimics the bioactivity of the myxoma virus protein M-T5 [197]. RRM was also used to design a negative 22 AA control peptide, RRM-C, which was not cytotoxic [197]. The M-T5 protein interacts with Akt (serine/threonine kinase protein) and this interaction regulates myxoma virus permissiveness in cells [198]. The cytotoxic effects of RRM-MV peptide was investigated on the mouse melanoma (B16-F10), human squamous cell carcinoma (Colo16), mouse macrophage (J774) and Chinese hamster ovary (CHO) cell lines. RRM-MV was only cytotoxic to B16-F10 and Colo16 cells while the negative peptide RRM-C was not [197]. Almansour et al. [199] also observed that RRM-MV was cytotoxic to human MM96L melanoma cells and Colo 16 squamous cell carcinoma cells but not epidermal melanocytes (HEM) or dermal fibroblasts (HDF) [199].

RRM was also employed to design a bioactive peptide possessing interleukin-12 (IL-12) activity. IL-12 is a heterodimeric pro-inflammatory cytokine that is produced by dendritic and phagocytic cells in response to pathogens during an infection [200]. IL-12 has anti-tumour activity in murine models of melanoma, lung, kidney, ovarian and colon cancers [200]. In total 13 IL-12 proteins from different species have been analysed using RRM [201]. The bioactive peptide analogue IL-12 devised from mouse was cytotoxic to mouse melanoma (B16-F10), mouse dermal fibroblasts, Chinese Hamster Ovary (CHO) and mouse macrophages (J774) [201], however the negative control peptide RRM-C was shown to be non-cytotoxic [201].

## Therapeutic peptides target tumour suppressor protein

p53 is often found mutated in many human cancers [202–204], the majority (75%) of which are missense mutations [205]. Other mutations include frameshifts (insertions or deletions) 9%, non-sense mutations 7% and silent mutations 5% [206]. Normally, endogenous p53 levels are low because of its rapid degradation by ubiquitin-dependent proteolysis [207]. Its levels rise in response to cellular stresses such as DNA damage [208] and if there is a significant damage then it promotes the cell to undergo apoptosis [209]. MDM2 binds to p53 and suppresses its activity as a transcription factor [210], it can also promote its rapid degradation [211]. Several studies have utilised different types of peptides derived from the amino group of p53 that were designed to block the interaction between MDM2 and p53 interaction, thereby preventing the rapid degradation of the latter in the cell. Bottger et al. [212] reported that the TIP peptide derived from the N-terminal MDM2-binding domain region of p53 can inhibit its interaction with MDM2. This led to an accumulation the p53 in the cell as well as the activation of the p53 transcription factor [212]. The peptide homologue of p53 disrupted the MDM2-p53 interaction in SA1 osteosarcoma cells that overexpresses MDM2 [213]. This caused in an increase in the level of p53 transcriptional activity and resulted in the inhibition of colony formation as well cell cycle arrest and increased levels of apoptosis [213].

Kanovsky et al. [214] synthesised three peptides from the MDM2-binding domain of p53, PNC-27 (12–26 AA), PNC-21 (12–20 AA) and PNC-28 (17–26 AA). These three peptides were attached to a cell penetrating sequence at their carboxyl terminus. All three peptides were cytotoxic against human metastatic colon adenocarcinoma cells, transformed rat brain capillary endothelial cells, human cervical carcinoma, human metastatic breast carcinoma cells, human non-small cell lung carcinoma and human osteosarcoma in vitro, but not to non-cancerous cells such as rat pancreatic acinar cells [214]. Do et al. [215] investigated the cytotoxicity of PNC-27 peptide on human breast cancer cell lines: MDA-MB-468 (mutant p53), MCF-7 (overexpressed wild type p53) and MDA-MB-157 (null p53). They found that PNC-27 induced necrosis in these breast cancer cells and this was p53-independent [215]. PNC-27 interacted with MDM2 in human leukaemia K562 cancer cells (p53 null) and caused pore formation resulting in cell death [216]. This peptide interacted with HDM2 that is highly expressed on the membrane of MIA-PaCa-2 human pancreatic cancer, MCF-7 breast cancer, A-2058 melanoma and TUC-3 Rat k-ras-transformed pancreatic cancer cells but not in normal cells [217]. This peptide also induced cell lysis in human breast cancer cells [218].

Bowne et al. [219] found that the CPP, penetratin which was conjugated in the carboxyl terminal end of the PNC-28 peptide induced necrosis in human pancreatic cancer cells. PNC-28 created pores in the cell membrane resulting in release of lactate dehydrogenase (LDH) [219]. When the penetratin sequence was removed from the PNC-28 peptide, human pancreatic cancer cells underwent apoptosis by inducing caspase-3 and −7 levels [219]. The anti-HMD2 peptides were generated by fusing Tat peptide to a p53-derived peptide that binds to HDM2 [220]. Tat-αHDM2 inhibited the interaction of p53 and HDM2, resulting in the death of several human cancer cell lines including: melanoma, retinoblastoma, osteosarcoma and cervical carcinoma [220]. In human osteosarcoma cells (U2OS), the addition of Tat-αHDM2 increased p53 levels which caused an activation of apoptotic genes (e.g., p21, Pig3, and Bax) and halted the cell from cycling [220].

## Therapeutic peptides target transcription factors

The Myc gene family encodes three transcription factors: c-Myc, N-Myc and L-Myc which are involved in regulation of various cellular processes such as cell growth and death, cell cycle, differentiation and transcription [221]. Deregulated Myc proteins are found in many tumours including breast [222], ovarian [223], small cell lung carcinoma [224], melanoma [225], cervical [226], multiple myeloma [227] and gastric cancer [228]. The Myc protein contains a basic region following by helix-loop-helix and a leucine zipper (BR-HLH-LZ) motifs which is a family of DNA-binding proteins [229]. Draeger and Mullen [18] used a 14 AA peptide (H1-S6A, F8A) derived from the helix 1 (H1) C-terminal region of c-Myc which inhibited c-Myc DNA binding. Giorello et al. [230] joined the H1-S6A, F8A peptide with the penetratin sequence from Antennapedia. This fused peptide, Int-H1-S6A, F8A, inhibited cell proliferation and induce apoptosis in MCF-7 human breast cancer cells as well as blocking the activity of c-Myc [230]. The penetratin peptide was fused with ELP and the c-Myc peptide inhibitor (H1-S6A, F8A), and was called Pen-ELP-H1 [76]. Pen-ELP-H1 inhibited the transcription of c-Myc genes that prevented the growth of MCF-7 human breast cancer cells in vitro. Hyperthermia enhanced the cellular uptake of this polypeptide by ~13-fold compared to that seen at 37 °C [76]. This is most likely due to the formation of aggregates that enhanced the uptake and delivery of this peptide to the tumour target site [76]. The ELP was fused into a CPP derived from bactenecin and c-Myc inhibitory peptide (H1-S6A,F8A), this peptide was called BAC-ELP-H1. This peptide inhibited proliferation in human and rat glioma cells in vitro and reduced tumour volume by 80% in rat glioma [231].

## Conclusion

In this review, we have highlighted the potential of using therapeutic peptides in treating cancers. Therapeutic peptides can be designed to target almost any protein of interest due to ease of synthesis and high target specificity and selectivity. Various therapeutic peptides have been selectively designed to target signal transduction pathways, cell cycle, tumour suppressor proteins as well as transcription factors. These therapeutic peptides bind specifically to those target protein to which they are designed for. They induce cell death in various cancer cells in vitro and in vivo. They show selectivity in targeting cancer cells without damaging untransformed cells. Although there are advantages of using peptide-based cancer therapy, it does have limitations. Various approaches have been used to overcome peptides limitations such as using CPP for efficient delivery of these anti-cancer peptides to their tumour cell targets. Therapeutic peptides represent a new and exciting approach for cancer therapy.

## Abbreviations

AA: Amino acids
CPP: Cell-penetrating peptide
Dox: Doxorubicin
ELP: Elastin-like polypeptides
ERK: Extracellular signal-regulated kinase
HDM2: Human double minute 2
JNK: c-Jun amino-terminal kinase
LPD: Liposome-polycation-DNA
MAPK: Mitogen activated protein kinase
MDM2: Murine double minute 2
MEKI: MEK inhibition
p38: P38 protein kinase
Penetratin: CPP from antennapedia
Rb: Retinoblastoma
RGD: Arginine -Glycine- Aspartic acid
siRNA: Small interfering RNA
Tat: CPP from HIV- transcription factor
TTP: Tumour-targeting peptides

## References

[1] Global Burden of Disease Cancer, C (2015). The Global Burden of Cancer 2013. JAMA Oncology.

[2] Torre LA (2015). Global cancer statistics, 2012. CA Cancer J Clin.

[3] Bray F, Moller B (2006). Predicting the future burden of cancer. Nat Rev Cancer.

[4] Vogelstein B (2013). Cancer genome landscapes. Science.

[5] Kurreck J, Stein CA (2016). Molecular Medicine: An Introduction.

[6] Burrell RA (2013). The causes and consequences of genetic heterogeneity in cancer evolution. Nature.

[7] Langley RR, Fidler IJ (2007). Tumor cell-organ microenvironment interactions in the pathogenesis of cancer metastasis. Endocr Rev.

[8] Biemar F, Foti M (2013). Global progress against cancer—challenges and opportunities. Cancer Biol Med.

[9] Mahassni SH, Al-Reemi RM (2013). Apoptosis and necrosis of human breast cancer cells by an aqueous extract of garden cress (Lepidium sativum) seeds. Saudi J Biol Sci.

[10] Ponnusamy L, Mahalingaiah PK, Singh KP (2016). Chronic Oxidative Stress Increases Resistance to Doxorubicin-Induced Cytotoxicity in Renal Carcinoma Cells Potentially Through Epigenetic Mechanism. Mol Pharmacol.

[11] Zhou S, Palmeira CM, Wallace KB (2001). Doxorubicin-induced persistent oxidative stress to cardiac myocytes. Toxicol Lett.

[12] Joshi G (2005). Free radical mediated oxidative stress and toxic side effects in brain induced by the anti cancer drug adriamycin: insight into chemobrain. Free Radic Res.

[13] Rivera E, Gomez H (2010). Chemotherapy resistance in metastatic breast cancer: the evolving role of ixabepilone. Breast Cancer Res.

[14] Eichler AF (2008). Survival in patients with brain metastases from breast cancer: the importance of HER-2 status. Cancer.

[15] Gerber B, Freund M, Reimer T (2010). Recurrent Breast Cancer: Treatment Strategies for Maintaining and Prolonging Good Quality of Life. Dtsch Arztebl Int.

[16] Hayashi MA, Ducancel F, Konno K (2012). Natural Peptides with Potential Applications in Drug Development, Diagnosis, and/or Biotechnology. Int J Pept.

[17] Bidwell GL, Raucher D (2009). Therapeutic peptides for cancer therapy. Part I - peptide inhibitors of signal transduction cascades. Expert Opin Drug Deliv.

[18] Draeger LJ, Mullen GP (1994). Interaction of the bHLH-zip domain of c-Myc with H1-type peptides. Characterization of helicity in the H1 peptides by NMR. J Biol Chem.

[19] Chen IT (1996). Characterization of p21Cip1/Waf1 peptide domains required for cyclin E/Cdk2 and PCNA interaction. Oncogene.

[20] Ali R, Rani R, Kumar S, Ghulam Md A, Ishfaq Ahmed S (2013). New Peptide Based Therapeutic Approaches. Advances in Protein Chemistry.

[21] Boohaker RJ (2012). The use of therapeutic peptides to target and to kill cancer cells. Curr Med Chem.

[22] McGregor DP (2008). Discovering and improving novel peptide therapeutics. Curr Opin Pharmacol.

[23] Cicero AF, Fogacci F, Colletti A (2017). Potential role of bioactive peptides in prevention and treatment of chronic diseases: a narrative review. Br J Pharmacol.

[24] Thundimadathil J (2012). Cancer Treatment Using Peptides: Current Therapies and Future Prospects. J Amino Acids.

[25] Craik DJ (2013). The future of peptide-based drugs. Chem Biol Drug Des.

[26] Blanco-Miguez A (2016). From amino acid sequence to bioactivity: The biomedical potential of antitumor peptides. Protein Sci.

[27] Gaspar D, Veiga AS, Castanho MA (2013). From antimicrobial to anticancer peptides. A review. Front Microbiol.

[28] Bowdish DM, Davidson DJ, Hancock RE (2006). Immunomodulatory properties of defensins and cathelicidins. Curr Top Microbiol Immunol.

[29] Lien S, Lowman HB (2003). Therapeutic peptides. Trends Biotechnol.

[30] Marr AK, Gooderham WJ, Hancock REW (2006). Antibacterial peptides for therapeutic use: obstacles and realistic outlook. Curr Opin Pharmacol.

[31] Zasloff M (1987). Magainins, a class of antimicrobial peptides from Xenopus skin: isolation, characterization of two active forms, and partial cDNA sequence of a precursor. Proc Natl Acad Sci U S A.

[32] Lehmann J (2006). Antitumor activity of the antimicrobial peptide magainin II against bladder cancer cell lines. Eur Urol.

[33] Cole AM, Weis P, Diamond G (1997). Isolation and characterization of pleurocidin, an antimicrobial peptide in the skin secretions of winter flounder. J Biol Chem.

[34] Hilchie AL (2011). Pleurocidin-family cationic antimicrobial peptides are cytolytic for breast carcinoma cells and prevent growth of tumor xenografts. Breast Cancer Res.

[35] Park CB, Kim MS, Kim SC (1996). A novel antimicrobial peptide from Bufo bufo gargarizans. Biochem Biophys Res Commun.

[36] Cho JH, Sung BH, Kim SC (2009). Buforins: histone H2A-derived antimicrobial peptides from toad stomach. Biochim Biophys Acta.

[37] Lee HS (2008). Mechanism of anticancer activity of buforin IIb, a histone H2A-derived peptide. Cancer Lett.

[38] Regberg J, Srimanee A, Langel Ü (2012). Applications of Cell-Penetrating Peptides for Tumor Targeting and Future Cancer Therapies. Pharm.

[39] Walrant A (2013). Direct translocation of cell-penetrating peptides in liposomes: A combined mass spectrometry quantification and fluorescence detection study. Anal Biochem.

[40] Drin G (2003). Studies on the internalization mechanism of cationic cell-penetrating peptides. J Biol Chem.

[41] Richard JP (2003). Cell-penetrating peptides. A reevaluation of the mechanism of cellular uptake. J Biol Chem.

[42] Fretz MM (2007). Temperature-, concentration- and cholesterol-dependent translocation of L- and D-octa-arginine across the plasma and nuclear membrane of CD34(+) leukaemia cells. Biochem J.

[43] Duchardt F (2007). A comprehensive model for the cellular uptake of cationic cell-penetrating peptides. Traffic.

[44] Fischer R (2004). A stepwise dissection of the intracellular fate of cationic cell-penetrating peptides. J Biol Chem.

[45] Vives E, Brodin P, Lebleu B (1997). A truncated HIV-1 Tat protein basic domain rapidly translocates through the plasma membrane and accumulates in the cell nucleus. J Biol Chem.

[46] Astriab-Fisher A (2002). Conjugates of antisense oligonucleotides with the Tat and antennapedia cell-penetrating peptides: effects on cellular uptake, binding to target sequences, and biologic actions. Pharm Res.

[47] Torchilin VP (2001). TAT peptide on the surface of liposomes affords their efficient intracellular delivery even at low temperature and in the presence of metabolic inhibitors. Proc Natl Acad Sci U S A.

[48] Lindgren M (2006). Overcoming methotrexate resistance in breast cancer tumour cells by the use of a new cell-penetrating peptide. Biochem Pharmacol.

[49] Meade BR, Dowdy SF (2008). Enhancing the cellular uptake of siRNA duplexes following noncovalent packaging with protein transduction domain peptides. Adv Drug Deliv Rev.

[50] Nakase I, Tanaka G, Futaki S (2013). Cell-penetrating peptides (CPPs) as a vector for the delivery of siRNAs into cells. Mol Biosyst.

[51] Nakase I (2012). Efficient Intracellular Delivery of Nucleic Acid Pharmaceuticals Using Cell-Penetrating Peptides. Acc Chem Res.

[52] Lim KJ (2013). A Cancer Specific Cell-Penetrating Peptide, BR2, for the Efficient Delivery of an scFv into Cancer Cells. PLoS One.

[53] Liang JF, Yang VC (2005). Synthesis of doxorubicin-peptide conjugate with multidrug resistant tumor cell killing activity. Bioorg Med Chem Lett.

[54] Wickham TJ (1993). Integrins alpha v beta 3 and alpha v beta 5 promote adenovirus internalization but not virus attachment. Cell.

[55] Chen X (2005). Integrin alpha v beta 3-targeted imaging of lung cancer. Neoplasia.

[56] Seftor RE (1992). Role of the alpha v beta 3 integrin in human melanoma cell invasion. Proc Natl Acad Sci U S A.

[57] Gladson CL, Cheresh DA (1991). Glioblastoma expression of vitronectin and the alpha v beta 3 integrin. Adhesion mechanism for transformed glial cells. J Clin Investig.

[58] Carreiras F (1996). Expression and localization of alpha v integrins and their ligand vitronectin in normal ovarian epithelium and in ovarian carcinoma. Gynecol Oncol.

[59] Pignatelli M (1992). Integrins and their accessory adhesion molecules in mammary carcinomas: loss of polarization in poorly differentiated tumors. Hum Pathol.

[60] Chen ZY (2012). Cyclic RGD peptide-modified liposomal drug delivery system: enhanced cellular uptake in vitro and improved pharmacokinetics in rats. Int J Nanomedicine.

[61] Xiong XB (2005). Intracellular delivery of doxorubicin with RGD-modified sterically stabilized liposomes for an improved antitumor efficacy: in vitro and in vivo. J Pharm Sci.

[62] Woodle MC (1995). Sterically Stabilized Liposome Therapeutics. Adv Drug Deliv Rev.

[63] Nohara S (2016). Aminopeptidase N (APN/CD13) as a target molecule for scirrhous gastric cancer. Clin Res Hepatol Gastroenterol.

[64] Ikeda N (2003). Clinical significance of aminopeptidase N/CD13 expression in human pancreatic carcinoma. Clin Cancer Res.

[65] Zhang Q (2015). Expression and clinical significance of aminopeptidase N/CD13 in non-small cell lung cancer. J Cancer Res Ther.

[66] Chen Y, Wu JJ, Huang L (2010). Nanoparticles targeted with NGR motif deliver c-myc siRNA and doxorubicin for anticancer therapy. Mol Ther.

[67] Dinca A, Chien WM, Chin MT (2016). Intracellular Delivery of Proteins with Cell-Penetrating Peptides for Therapeutic Uses in Human Disease. Int J Mol Sci.

[68] Kwon MK (2008). Antitumor effect of a transducible fusogenic peptide releasing multiple proapoptotic peptides by caspase-3. Mol Cancer Ther.

[69] Yang H (2010). Chondroitin Sulfate as a Molecular Portal That Preferentially Mediates the Apoptotic Killing of Tumor Cells by Penetratin-directed Mitochondria-disrupting Peptides. J Biol Chem.

[70] Fu B (2014). Identification and characterization of a novel phage display-derived peptide with affinity for human brain metastatic breast cancer. Biotechnol Lett.

[71] Fu B (2015). Enhanced antitumor effects of the BRBP1 compound peptide BRBP1-TAT-KLA on human brain metastatic breast cancer. Sci Rep.

[72] Bidwell GL, Raucher D (2010). Cell penetrating elastin-like polypeptides for therapeutic peptide delivery. Adv Drug Deliv Rev.

[73] MacKay JA (2009). Self-assembling chimeric polypeptide-doxorubicin conjugate nanoparticles that abolish tumours after a single injection. Nat Mater.

[74] Massodi I, Bidwell GL, Raucher D (2005). Evaluation of cell penetrating peptides fused to elastin-like polypeptide for drug delivery. J Control Release.

[75] Massodi I (2010). Inhibition of ovarian cancer cell proliferation by a cell cycle inhibitory peptide fused to a thermally responsive polypeptide carrier. Int J Cancer.

[76] Bidwell GL, Raucher D (2005). Application of thermally responsive polypeptides directed against c-Myc transcriptional function for cancer therapy. Mol Cancer Ther.

[77] Conrad U (2011). ELPylated anti-human TNF therapeutic single-domain antibodies for prevention of lethal septic shock. Plant Biotechnol J.

[78] Shamji MF (2008). Synthesis and characterization of a thermally-responsive tumor necrosis factor antagonist. J Control Release.

[79] Chen TH, Bae Y, Furgeson DY (2008). Intelligent biosynthetic nanobiomaterials (IBNs) for hyperthermic gene delivery. Pharm Res.

[80] Raucher D, Massodi I, Bidwell GL (2008). Thermally targeted delivery of chemotherapeutics and anti-cancer peptides by elastin-like polypeptide. Expert Opinion on Drug Delivery.

[81] Meyer DE (2001). Targeting a genetically engineered elastin-like polypeptide to solid tumors by local hyperthermia. Cancer Res.

[82] Massodi I (2009). Inhibition of ovarian cancer cell metastasis by a fusion polypeptide Tat-ELP. Clin Exp Metastasis.

[83] Gottesman MM (2002). Mechanisms of cancer drug resistance. Annu Rev Med.

[84] Fojo AT, Menefee M (2005). Microtubule targeting agents: basic mechanisms of multidrug resistance (MDR). Semin Oncol.

[85] Bidwell GL (2007). A thermally targeted elastin-like polypeptide-doxorubicin conjugate overcomes drug resistance. Investig New Drugs.

[86] Bidwell GL (2007). Development of elastin-like polypeptide for thermally targeted delivery of doxorubicin. Biochem Pharmacol.

[87] Penchala SC (2015). A biomimetic approach for enhancing the in vivo half-life of peptides. Nat Chem Biol.

[88] Roberts MJ, Bentley MD, Harris JM (2012). Chemistry for peptide and protein PEGylation. Adv Drug Deliv Rev.

[89] Garay RP (2012). Antibodies against polyethylene glycol in healthy subjects and in patients treated with PEG-conjugated agents. Expert Opinion on Drug Delivery.

[90] Schellenberger V (2009). A recombinant polypeptide extends the in vivo half-life of peptides and proteins in a tunable manner. Nat Biotechnol.

[91] Podust VN (2013). Extension of in vivo half-life of biologically active peptides via chemical conjugation to XTEN protein polymer. Protein Eng Des Sel.

[92] Marier JF (2008). Pharmacokinetics, safety, and tolerability of teduglutide, a glucagon-like peptide-2 (GLP-2) analog, following multiple ascending subcutaneous administrations in healthy subjects. J Clin Pharmacol.

[93] Alters SE (2012). GLP2-2G-XTEN: A Pharmaceutical Protein with Improved Serum Half-Life and Efficacy in a Rat Crohn’s Disease Model. PLoS One.

[94] Xiao JP, Burn A, Tolbert TJ (2008). Increasing solubility of proteins and peptides by site-specific modification with betaine. Bioconjug Chem.

[95] Hao XY (2015). TAT Modification of Alpha-Helical Anticancer Peptides to Improve Specificity and Efficacy. PLoS One.

[96] Raucher D (2009). Therapeutic peptides for cancer therapy. Part II - cell cycle inhibitory peptides and apoptosis-inducing peptides. Expert Opin Drug Deliv.

[97] Hughes PE, Caenepeel S, Wu LC (2016). Targeted Therapy and Checkpoint Immunotherapy Combinations for the Treatment of Cancer. Trends Immunol.

[98] Gao B, Roux PP (2015). Translational control by oncogenic signaling pathways. Biochim Biophys Acta.

[99] Hoshino R (1999). Constitutive activation of the 41-/43-kDa mitogen-activated protein kinase signaling pathway in human tumors. Oncogene.

[100] Liu YS, Shepherd EG, Nelin LD (2007). MAPK phosphatases - regulating the immune response. Nat Rev Immunol.

[101] Meloche S, Pouyssegur J (2007). The ERK1/2 mitogen-activated protein kinase pathway as a master regulator of the G1- to S-phase transition. Oncogene.

[102] Dhillon AS (2007). MAP kinase signalling pathways in cancer. Oncogene.

[103] Santarpia L, Lippman SM, El-Naggar AK (2012). Targeting the MAPK-RAS-RAF signaling pathway in cancer therapy. Expert Opin Ther Targets.

[104] Prior IA, Lewis PD, Mattos C (2012). A comprehensive survey of Ras mutations in cancer. Cancer Res.

[105] Downward J. Targeting RAS signalling pathways in cancer therapy. Nat Rev Cancer. 2003;3(1):11–22. 10.1038/nrc969 12509763

[106] Pincus MR, Pincus MR (2011). Oncoproteins and early tumor detection. Henry’s Clinical Diagnosis and Management by Laboratory Methods.

[107] Lee G, Ronai ZA, Pincus MR, Murphy RB, Delohery TM, Nishimura S, Yamaizumi Z, Weinstein IB, Brandt-Rauf PW (1990). Inhibition of ras oncogene-encoded P21 protein-induced pinocytotic activity by a synthetic peptide corresponding to an effector domain of the protein. Med Sci Res.

[108] Kanovsky M (2003). Peptides designed from molecular modeling studies of the ras-p21 protein induce phenotypic reversion of a pancreatic carcinoma cell line but have no effect on normal pancreatic acinar cell growth. Cancer Chemother Pharmacol.

[109] Adler V (2008). Two peptides derived from ras-p21 induce either phenotypic reversion or tumor cell necrosis of ras-transformed human cancer cells. Cancer Chemother Pharmacol.

[110] Wu D (2014). Peptide-based cancer therapy: Opportunity and challenge. Cancer Lett.

[111] Vesely DL (2007). Elimination of up to 80% of human pancreatic adenocarcinomas in athymic mice by cardiac hormones. In Vivo.

[112] Vesely BA (2006). Urodilatin and four cardiac hormones decrease human renal carcinoma cell numbers. Eur J Clin Investig.

[113] Vesely BA (2007). Four cardiac hormones cause cell death in 81% of human ovarian adenocarcinoma cells. Cancer Therapy.

[114] Vesely BA (2007). Four cardiac hormones cause cell death of melanoma cells and inhibit their DNA synthesis. Am J Med Sci.

[115] Vesely BA (2007). Four cardiac hormones eliminate 4-fold more human glioblastoma cells than the green mamba snake peptide. Cancer Lett.

[116] Vesely BA (2006). Vessel dilator: most potent of the atrial natriuretic peptides in decreasing the number and DNA synthesis of human squamous lung cancer cells. Cancer Lett.

[117] Vesely BA (2005). Four peptide hormones decrease the number of human breast adenocarcinoma cells. Eur J Cancer.

[118] Eichelbaum EJ (2006). Four cardiac hormones eliminate up to 82% of human medullary thyroid carcinoma cells within 24 hour. Endocrine.

[119] Sun Y (2007). Insulin and Epidermal Growth Factor’s Activation of Extracellular-Signal Regulated Kinases 1/2 and DNA Synthesis Is Inhibited by Four Cardiac Hormones. J Cancer Mol.

[120] Sun Y (2009). Vessel dilator and kaliuretic peptide inhibit Ras in human prostate cancer cells. Anticancer Res.

[121] Sun Y (2009). Atrial natriuretic peptide and long-acting natriuretic peptide inhibit ras in human prostate cancer cells. Anticancer Res.

[122] Sun Y (2007). Atrial natriuretic peptide and long acting natriuretic peptide inhibit MEK 1/2 activation in human prostate cancer cells. Anticancer Res.

[123] Sun Y (2006). Atrial natriuretic peptide and long acting natriuretic peptide inhibit ERK 1/2 in prostate cancer cells. Anticancer Res.

[124] McCubrey JA (2007). Roles of the Raf/MEK/ERK pathway in cell growth, malignant transformation and drug resistance. Biochim Biophys Acta.

[125] Li XX (2013). Synthesis and Biological Evaluation of RGD-Conjugated MEK1/2 Kinase Inhibitors for Integrin-Targeted Cancer Therapy. Molecules.

[126] Hou J (2016). RGD peptide conjugation results in enhanced antitumor activity of PD0325901 against glioblastoma by both tumor-targeting delivery and combination therapy. Int J Pharm.

[127] Licato LL, Brenner DA (1998). Analysis of signaling protein kinases in human colon or colorectal carcinomas. Dig Dis Sci.

[128] Hirano T (2002). Dominant negative MEKK1 inhibits survival of pancreatic cancer cells. Oncogene.

[129] Wang HY, Cheng ZY, Malbon CC (2003). Overexpression of mitogen-activated protein kinase phosphatases MKP1, MKP2 in human breast cancer. Cancer Lett.

[130] Czibere A (2006). Exisulind induces apoptosis in advanced myelodysplastic syndrome (MDS) and acute myeloid leukaemia/MDS. Br J Haematol.

[131] Gross ND (2007). Inhibition of jun NH2-terminal kinases suppresses the growth of experimental head and neck squamous cell carcinoma. Clin Cancer Res.

[132] Maeda S (2008). S2052 c-Jun Nh2-terminal kinase 1 is a critical regulator for the development of chemical-induced gastric cancer in mice. Gastroenterology.

[133] Li JY (2008). Constitutive activation of c-Jun N-terminal kinase correlates with histologic grade and EGFR expression in diffuse gliomas. J Neuro-Oncol.

[134] Kaoud TS (2011). Development of JNK2-selective peptide inhibitors that inhibit breast cancer cell migration. ACS Chem Biol.

[135] Gill K (2012). Quantification of p38alphaMAP kinase: a prognostic marker in HNSCC with respect to radiation therapy. Clin Chim Acta.

[136] Davidson B (2006). The mitogen-activated protein kinases (MAPK) p38 and JNK are markers of tumor progression in breast carcinoma. Gynecol Oncol.

[137] Liang B (2005). Increased expression of mitogen-activated protein kinase and its upstream regulating signal in human gastric cancer. World J Gastroenterol.

[138] Greenberg AK (2002). Selective p38 activation in human non-small cell lung cancer. Am J Respir Cell Mol Biol.

[139] Dietrich J, Hulme C, Hurley LH (2010). The design, synthesis, and evaluation of 8 hybrid DFG-out allosteric kinase inhibitors: A structural analysis of the binding interactions of Gleevec®, Nexavar®, and BIRB-796. Bioorg Med Chem.

[140] Gill K (2013). Development of peptide inhibitor as a therapeutic agent against head and neck squamous cell carcinoma (HNSCC) targeting p38alpha MAP kinase. Biochim Biophys Acta.

[141] Gill K (2014). The rational design of specific peptide inhibitor against p38alpha MAPK at allosteric-site: a therapeutic modality for HNSCC. PLoS One.

[142] Suryadinata R, Sadowski M, Sarcevic B (2010). Control of cell cycle progression by phosphorylation of cyclin-dependent kinase (CDK) substrates. Biosci Rep.

[143] Malumbres M, Barbacid M (2005). Mammalian cyclin-dependent kinases. Trends Biochem Sci.

[144] Matsushime H (1992). Identification and properties of an atypical catalytic subunit (p34PSK-J3/cdk4) for mammalian D type G1 cyclins. Cell.

[145] Hwang HC, Clurman BE (2005). Cyclin E in normal and neoplastic cell cycles. Oncogene.

[146] Pagano M (1992). Cyclin A is required at two points in the human cell cycle. EMBO J.

[147] Hardwick LJA, Philpott A (2014). Nervous decision-making: to divide or differentiate. Trends Genet.

[148] Lim SH, Kaldis P (2013). Cdks, cyclins and CKIs: roles beyond cell cycle regulation. Development.

[149] Sherr CJ, Roberts JM (1999). CDK inhibitors: positive and negative regulators of G(sub.1)-phase progression. (first gap phase)(ST). Genes Dev.

[150] Sherr CJ, Beach D, Shapiro GI (2016). Targeting CDK4 and CDK6: From Discovery to Therapy. Cancer Discov.

[151] Liggett WH, Sidransky D (1998). Role of the p16 tumor suppressor gene in cancer. J Clin Oncol.

[152] Milde-Langosch K (2001). Overexpression of the p16 cell cycle inhibitor in breast cancer is associated with a more malignant phenotype. Breast Cancer Res Treat.

[153] Remo A (2016). p16 Expression in Prostate Cancer and Nonmalignant Lesions: Novel Findings and Review of the Literature. Appl Immunohistochem Mol Morphol.

[154] Mihic-Probst D (2006). p16 expression in primary malignant melanoma is associated with prognosis and lymph node status. Int J Cancer.

[155] Fischer CA (2010). p16 expression in oropharyngeal cancer: its impact on staging and prognosis compared with the conventional clinical staging parameters. Ann Oncol.

[156] Lesnikova I (2009). p16 as a diagnostic marker of cervical neoplasia: a tissue microarray study of 796 archival specimens. Diagn Pathol.

[157] Lam AKY (2008). p16 expression in colorectal adenocarcinoma: marker of aggressiveness and morphological types. Pathology.

[158] Gao YS, Li LZ, Song LJ (2015). Expression of p16 and Survivin in gliomas and their correlation with cell proliferation. Oncol Lett.

[159] Chintala SK (1997). Adenovirus-mediated p16/CDKN2 gene transfer suppresses glioma invasion in vitro. Oncogene.

[160] Steiner MS (2000). Adenoviral vector containing wild-type p16 suppresses prostate cancer growth and prolongs survival by inducing cell senescence. Cancer Gene Ther.

[161] Hung KS (2000). Expression of p16(INK4A) induces dominant suppression of glioblastoma growth in situ through necrosis and cell cycle arrest. Biochem Biophys Res Commun.

[162] Zhang X (2007). Effects of exogenous p16(ink4a) gene on biological behaviors of human lung cancer cells. J Huazhong Univ Sci Technolog Med Sci.

[163] Li L, Yang T (2003). Expression of Cdk4, Cyclin D1 and Rb in exogenous wild type P16 gene transfected bladder cancer cell line. Colloids Surf B Biointerfaces.

[164] Fahraeus R (1996). Inhibition of pRb phosphorylation and cell-cycle progression by a 20-residue peptide derived from p16CDKN2/INK4A. Curr Biol.

[165] Fahraeus R (1998). Characterization of the cyclin-dependent kinase inhibitory domain of the INK4 family as a model for a synthetic tumour suppressor molecule. Oncogene.

[166] Fujimoto K (2000). Inhibition of pRb phosphorylation and cell cycle progression by an antennapedia-p16INK4A fusion peptide in pancreatic cancer cells. Cancer Lett.

[167] Hosotani R (2002). Trojan p16 Peptide Suppresses Pancreatic Cancer Growth and Prolongs Survival in Mice. Clin Cancer Res.

[168] Cazzalini O (2010). Multiple roles of the cell cycle inhibitor p21(CDKN1A) in the DNA damage response. Mutat Res.

[169] Abbas T, Dutta A (2009). p21 in cancer: intricate networks and multiple activities. Nat Rev Cancer.

[170] Luo Y, Hurwitz J, Massague J (1995). Cell-cycle inhibition by independent CDK and PCNA binding domains in p21Cip1. Nature.

[171] Warbrick E (1995). A small peptide inhibitor of DNA replication defines the site of interaction between the cyclin-dependent kinase inhibitor p21WAF1 and proliferating cell nuclear antigen. Curr Biol.

[172] Pan ZQ (1995). Inhibition of Nucleotide Excision-Repair by the Cyclin-Dependent Kinase Inhibitor P21. J Biol Chem.

[173] Ball KL (1997). Cell-cycle arrest and inhibition of Cdk4 activity by small peptides based on the carboxy-terminal domain of p21WAF1. Curr Biol.

[174] Cayrol C, Knibiehler M, Ducommun B (1998). p21 binding to PCNA causes G1 and G2 cell cycle arrest in p53-deficient cells. Oncogene.

[175] Mutoh M (1999). A p21Waf1/Cip1 Carboxyl-terminal Peptide Exhibited Cyclin-dependent Kinase-inhibitory Activity and Cytotoxicity When Introduced into Human Cells. Cancer Res.

[176] Mikecin AM (2014). Thermally targeted p21 peptide enhances bortezomib cytotoxicity in androgen-independent prostate cancer cell lines. Anticancer Drugs.

[177] Elmore S (2007). Apoptosis: a review of programmed cell death. Toxicol Pathol.

[178] Wong RS (2011). Apoptosis in cancer: from pathogenesis to treatment. J Exp Clin Cancer Res.

[179] Brunelle JK, Letai A (2009). Control of mitochondrial apoptosis by the Bcl-2 family. J Cell Sci.

[180] Gross A, McDonnell JM, Korsmeyer SJ (1999). BCL-2 family members and the mitochondria in apoptosis. Genes Dev.

[181] Yip KW, Reed JC (2008). Bcl-2 family proteins and cancer. Oncogene.

[182] Burlacu A (2003). Regulation of apoptosis by Bcl-2 family proteins. J Cell Mol Med.

[183] Westphal D (2011). Molecular biology of Bax and Bak activation and action. BBA-Mol Cell Res.

[184] Youle RJ, Strasser A (2008). The BCL-2 protein family: opposing activities that mediate cell death. Nat Rev Mol Cell Biol.

[185] Riedl SJ, Salvesen GS (2007). The apoptosome: signalling platform of cell death. Nat Rev Mol Cell Biol.

[186] Raffo AJ (1995). Overexpression of bcl-2 protects prostate cancer cells from apoptosis in vitro and confers resistance to androgen depletion in vivo. Cancer Res.

[187] Castle VP (1993). Expression of the Apoptosis-Suppressing Protein Bcl-2, in Neuroblastoma Is Associated with Unfavorable Histology and N-Myc Amplification. Am J Pathol.

[188] Gobe G (2002). Apoptosis and expression of Bcl-2, Bcl-XL, and Bax in renal cell carcinomas. Cancer Investig.

[189] Del Bufalo D (1997). Bcl-2 overexpression enhances the metastatic potential of a human breast cancer line. FASEB J.

[190] Coustan-Smith E (1996). Clinical relevance of BCL-2 overexpression in childhood acute lymphoblastic leukemia. Blood.

[191] Majid A (2008). BCL2 expression in chronic lymphocytic leukemia: lack of association with the BCL2 938A > C promoter single nucleotide polymorphism. Blood.

[192] Kondo E (1994). Expression of Bcl-2 protein and Fas antigen in non-Hodgkin’s lymphomas. Am J Pathol.

[193] Kashiwagi H (2007). TAT-Bim induces extensive apoptosis in cancer cells. Ann Surg Oncol.

[194] Valero JG (2011). Bax-derived membrane-active peptides act as potent and direct inducers of apoptosis in cancer cells. J Cell Sci.

[195] Boohaker RJ (2012). Rational development of a cytotoxic peptide to trigger cell death. Mol Pharm.

[196] Lee MW (2014). The CT20 peptide causes detachment and death of metastatic breast cancer cells by promoting mitochondrial aggregation and cytoskeletal disruption. Cell Death Dis.

[197] Istivan TS (2011). Biological effects of a de novo designed myxoma virus peptide analogue: evaluation of cytotoxicity on tumor cells. PLoS One.

[198] Wang G (2006). Infection of human cancer cells with myxoma virus requires Akt activation via interaction with a viral ankyrin-repeat host range factor. Proc Natl Acad Sci U S A.

[199] Almansour NM (2012). A bioactive peptide analogue for myxoma virus protein with a targeted cytotoxicity for human skin cancer in vitro. J Biomed Sci.

[200] Trinchieri G (2003). Interleukin-12 and the regulation of innate resistance and adaptive immunity. Nat Rev Immunol.

[201] Pirogova E (2011). Advances in methods for therapeutic peptide discovery, design and development. Curr Pharm Biotechnol.

[202] Ecke TH (2010). TP53 gene mutations in prostate cancer progression. Anticancer Res.

[203] Kandoth C (2013). Mutational landscape and significance across 12 major cancer types. Nature.

[204] Mogi A, Kuwano H (2011). TP53 mutations in nonsmall cell lung cancer. J Biomed Biotechnol.

[205] Freed-Pastor WA, Prives C (2012). Mutant p53: one name, many proteins. Genes Dev.

[206] Soussi T (2007). p53 alterations in human cancer: more questions than answers. Oncogene.

[207] Maki CG, Huibregtse JM, Howley PM (1996). In vivo ubiquitination and proteasome-mediated degradation of p53. Cancer Res.

[208] Lakin ND, Jackson SP (1999). Regulation of p53 in response to DNA damage. Oncogene.

[209] Fridman JS, Lowe SW (2003). Control of apoptosis by p53. Oncogene.

[210] Oliner JD (1993). Oncoprotein MDM2 conceals the activation domain of tumour suppressor p53. Nature.

[211] Haupt Y (1997). Mdm2 promotes the rapid degradation of p53. Nature.

[212] Böttger A (1997). Design of a synthetic Mdm2-binding mini protein that activates the p53 response in vivo. Curr Biol.

[213] Wasylyk C (1999). p53 mediated death of cells overexpressing MDM2 by an inhibitor of MDM2 interaction with p53. Oncogene.

[214] Kanovsky M (2001). Peptides from the amino terminal mdm-2-binding domain of p53, designed from conformational analysis, are selectively cytotoxic to transformed cells. Proc Natl Acad Sci U S A.

[215] Do TN (2003). Preferential induction of necrosis in human breast cancer cells by a p53 peptide derived from the MDM2 binding site. Oncogene.

[216] Davitt K (2014). The Anti-Cancer Peptide, PNC-27, Induces Tumor Cell Necrosis of a Poorly Differentiated Non-Solid Tissue Human Leukemia Cell Line that Depends on Expression of HDM-2 in the Plasma Membrane of these Cells. Ann Clin Lab Sci.

[217] Sarafraz-Yazdi E (2010). Anticancer peptide PNC-27 adopts an HDM-2-binding conformation and kills cancer cells by binding to HDM-2 in their membranes. Proc Natl Acad Sci U S A.

[218] Sookraj KA (2010). The anti-cancer peptide, PNC-27, induces tumor cell lysis as the intact peptide. Cancer Chemother Pharmacol.

[219] Bowne WB (2008). The Penetratin Sequence in the Anticancer PNC-28 Peptide Causes Tumor Cell Necrosis Rather Than Apoptosis of Human Pancreatic Cancer Cells. Ann Surg Oncol.

[220] Harbour JW (2002). Transducible peptide therapy for uveal melanoma and retinoblastoma. Arch Ophthalmol.

[221] Vita M, Henriksson M (2006). The Myc oncoprotein as a therapeutic target for human cancer. Semin Cancer Biol.

[222] Xu J, Chen Y, Olopade OI (2010). MYC and Breast Cancer. Genes Cancer.

[223] Wu R (2003). Amplification and overexpression of the L-MYC proto-oncogene in ovarian carcinomas. Am J Pathol.

[224] Lui WO, Tanenbaum DM, Larsson C (2001). High level amplification of 1p32-33 and 2p22-24 in small cell lung carcinomas. Int J Oncol.

[225] Treszl A (2004). Extra copies of c-myc are more pronounced in nodular melanomas than in superficial spreading melanomas as revealed by fluorescence in situ hybridisation. Cytometry B Clin Cytom.

[226] Abba MC (2004). The c-myc activation in cervical carcinomas and HPV 16 infections. Mutat Res.

[227] Avet-Loiseau H (2001). Rearrangements of the c-myc oncogene are present in 15% of primary human multiple myeloma tumors. Blood.

[228] de Souza CR (2013). MYC deregulation in gastric cancer and its clinicopathological implications. PLoS One.

[229] Tansey WP. Mammalian MYC Proteins and Cancer. New J Sci. 2014;2014:757534.

[230] Giorello L (1998). Inhibition of cancer cell growth and c-Myc transcriptional activity by a c-Myc helix 1-type peptide fused to an internalization sequence. Cancer Res.

[231] Bidwell GL (2013). Thermally targeted delivery of a c-Myc inhibitory polypeptide inhibits tumor progression and extends survival in a rat glioma model. PLoS One.

[232] Michl J (2006). PNC-28, a p53-derived peptide that is cytotoxic to cancer cells, blocks pancreatic cancer cell growth in vivo. Int J Cancer.

